# *TRPC6* Deletion Enhances eNOS Expression and Reduces LPS—Induced Acute Lung Injury

**DOI:** 10.3390/ijms242316756

**Published:** 2023-11-25

**Authors:** Mengyuan Wang, Xingfang Zhang, Juan Guo, Shangze Yang, Fang Yang, Xingjuan Chen

**Affiliations:** 1 Institute of Medical Research, Northwestern Polytechnical University, Xi’an 710072, China; ys211055001378@qhu.edu.cn (M.W.);; 2 Department of Pharmacy, Faculty of Medicine, Qinghai University, Xining 810001, China; xingfang_zhang@foxmail.com (X.Z.);

**Keywords:** acute lung injury, TRPC6, eNOS, endothelial cells

## Abstract

Acute lung injury (ALI) is characterized by endothelial barrier disruption and associated inflammatory responses, and transient receptor potential cation channel 6 (TRPC6)—mediated Ca^2+^ influx is critical for endothelial hyperpermeability. In this study, we investigated the role of TRPC6 in LPS—induced ALI, analyzed gene expression in *WT* and *TRPC6^-/-^* lungs using RNA sequencing, and explored the effects of TRPC6 in the LPS—induced hyperpermeability in human umbilical vein endothelial cells (HUVECs) to elucidate the underlying mechanisms. Intratracheal instillation of LPS caused edema in the mouse lungs. Deletion of TRPC6 reduced LPS—induced lung edema and decreased cell infiltration. RNA sequencing analysis suggested that downregulated cell adhesion molecules in *TRPC6^-/-^* lungs may be responsible for their resistance to LPS—induced injury. In addition, downregulation of TRPC6 significantly alleviated the LPS—induced decrease in eNOS expression in lung tissue as well as in HUVECs. Moreover, inhibition of TRPC6 with the channel antagonist larixyl led to a decrease in LPS—induced hyperpermeability and ROS production in HUVECs, which could be reversed by blocking eNOS. Our findings suggest that inhibition of TRPC6 ameliorates LPS—induced ALI, which may be achieved by acting on the cell adhesion molecule signaling pathway and participating in the regulation of eNOS levels in endothelial cells.

## 1. Introduction

Acute lung injury (ALI) is a severe pulmonary disease characterized by excessive activation of pulmonary inflammatory responses [1] and subsequent damage to lung tissues [2]. ALI can progress further to acute respiratory distress syndrome, a leading cause of death in critically ill patients [3]. However, the pathophysiological process of ALI is an intricate and multifaceted phenomenon involving complex interactions among various cell types and molecular pathways [4]. No effective therapies have been found for treating ALI/ARDS. Thus, there is an urgent need to reveal the pathophysiological mechanisms underlying ALI and effective treatment methods.

Studies have indicated that pulmonary microvascular endothelial cells (ECs) are crucial in the development of ALI [5,6,7]. The structure and function of ECs are finely regulated by calcium ion channels [8,9]. It is known that calcium entry into ECs is essential for barrier disruption [10]. Targeting the Ca^2+^ machinery is considered as a potential strategy to protect the endothelial barrier and improve ALI treatment outcomes. Among the identified calcium dependent—molecular mechanisms in ALI, endothelial nitric oxide synthase (eNOS) uncoupling plays an important role in inflammasome activation during lipopolysaccharide (LPS)—mediated ALI [11].

TRPC6 is a member of the TRP family and regulates Ca^2+^ influx into non—excitable cells [12]. Recent studies have found that TRPC6 may play a significant regulatory role in the development of ALI [13]. It has been recognized that TRPC6—dependent Ca^2+^ influx into ECs is a secondary response to TLR4 stimulation, contributing to lipopolysaccharide (LPS)—induced lung vascular barrier disruption and inflammation [14]. Activation of TRPC6 channels leads to calcium influx, promoting infiltration of inflammatory cells, increasing endothelial cell permeability, and participating in lung epithelial cell injury and repair [12]. The mechanism underlying overactivated TRPC6 contributing to LPS—induced ALI remains obscure. In this study, we aimed to investigate the potential impact of TRPC6 deletion in LPS—induced ALI using *TRPC6^-/-^* mice and found that TRPC6 deletion may enhance eNOS expression, which could account for its resistance against LPS—induced injury.

## 2. Results

### 2.1. Knockout of the TRPC6 Alleviates Acute Lung Inflammation in Lps—Treated Mice

**Figure 1:**
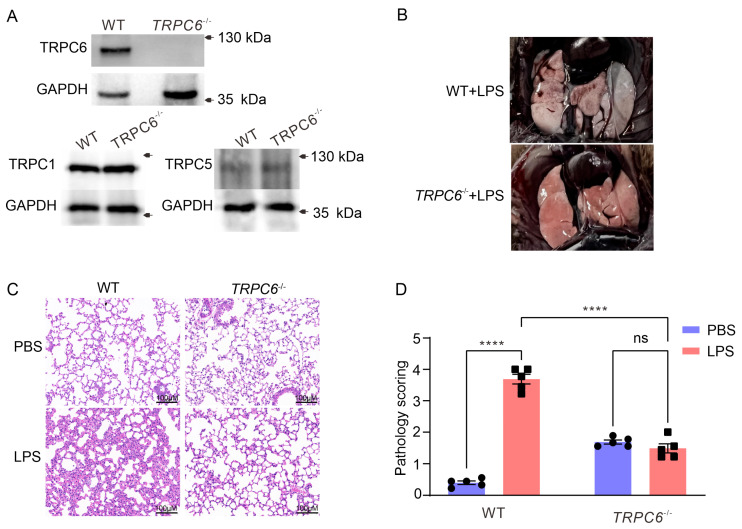
TRPC6 deletion improves the pulmonary histopathological changes induced by LPS. (**A**) Western blot analysis was performed to determine the expression levels of TRPC1, TRPC5, and TRPC6 in lung tissue lysates from *WT* and *TRPC6^-/-^* mice. (**B**) Representative images of lung tissue from *WT* and *TRPC6^-/-^* mice (*n* = 5 per group) at 24 h after LPS administration are shown. Evans Blue was injected via the tail vein 10 min before mice were sacrificed. (**C**) Representative images of H&E—stained lung sections from *WT* and *TRPC6^-/-^* mice (*n* = 5 per group) at 24 h after LPS administration, demonstrating alveolar structure and leukocyte infiltration. Scale bar, 100 μm. (**D**) Lung histopathological scores are presented as mean ± SEM; **** indicates *p* < 0.0001, ns—No significance.

Lung tissues were collected from wild—type (*WT*) and *TRPC6^-/-^* male mice. Immunoblot analysis was performed to assess the expression of TRPC1, TRPC5, and TRPC6 in lung tissue. As depicted in Figure 1A, complete ablation of TRPC6 was observed in *TRPC6^-/-^* mice without any impact on the expression levels of TRPC1 and TRPC5. A significant leakage of Evans Blue from the lungs was observed in *WT* mice treated with LPS, whereas *TRPC6^-/-^* mice exhibited reduced permeability (Figure 1B). To further assess whether *TRPC6* knockout reduced lung inflammation in LPS—treated mice, we examined lung tissue sections using H&E. As shown in Figure 1C,D, LPS treatment caused alveolar distortion, hemorrhage, and immune cell infiltration in the lung sections of *WT* mice, whereas these histological changes were attenuated in *TRPC6^-/-^* mice. Compared to the *WT*—PBS group, the lung injury score was significantly increased in the *WT*—LPS group (Figure 1D, 0.40 ± 0.06 vs. 3.69 ± 0.16, *n* = 5, **** *p* < 0.0001), while there was no significant difference between the *TRPC6^-/-^*—PBS and *TRPC6^-/-^*—LPS groups (Figure 1D, 1.69 ± 0.065 vs. 1.49 ± 0.14, *n* = 5, *p* = 0.24). These data indicated that knockout of TRPC6 improves LPS—induced histopathological changes in male mice.

**Figure 2:**
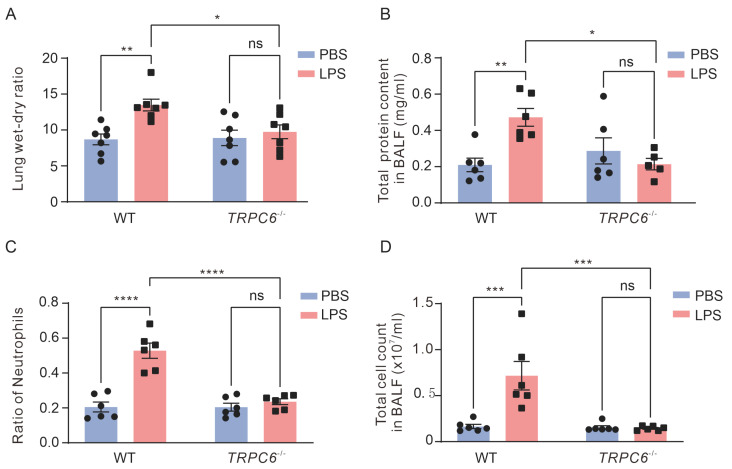
Deletion of the *TRPC6* alleviated lung injury in male mice with ALI. (**A**) Lung wet—to—dry weight ratio in male mice. (**B**) Total protein content in BALF in male mice. (**C**) The ratio of neutrophil count in BALF in male mice. (**D**) Total cell counts in BALF in male mice. (Plot shows mean ± SEM, * *p* < 0.05; ** *p* < 0.01; *** *p* < 0.001; **** *p* < 0.0001, ns—No significance).

Compared to the control mice, LPS treatment significantly exacerbated lung damage, as evidenced by increased lung wet—to—dry weight ratio (Figure 2A, 8.69 ± 0.75 vs. 13.48 ± 0.82, *n* = 7, ** *p* < 0.01), protein concentration (Figure 2B, 0.21 ± 0.04 vs. 0.47 ± 0.05 mg/mL, *n* = 6, ** *p* < 0.01), the ratio of neutrophil count (Figure 2C, 0.21 ± 0.03 vs. 0.53 ± 0.04, *n* = 6, **** *p* < 0.0001), and total cell count (Figure 2D, 1.65 ± 0.23 × 10^6^ vs. 7.17 ± 1.54 × 10^6^, *n* = 6, *** *p* < 0.001) in BALF. These findings indicate impaired pulmonary endothelial barrier function due to edema caused by intratracheal instillation of LPS. *TRPC6^-/-^* mice treated with LPS did not exhibit an increase in lung wet—to—dry weight ratio (Figure 2A, 10.00 ± 0.94 vs 9.72 ± 0.75., *n* = 7–8, *p* = 0.94), protein concentration (Figure 2B, 0.21 ± 0.03 vs. 0.29 ± 0.07 mg/mL, *n* = 6, *p* = 0.76), the ratio of neutrophil count in BALF (Figure 2C, 0.24 ± 0.02 vs 0.20 ± 0.02., *n* = 6, *p* = 0.87), and total cell count (Figure 2D, 1.54 ± 0.19 × 10^6^ vs. 1.44 ± 0.01 × 10^6^, *n* = 6, *p* = 0.99). Therefore, the knockout of *TRPC6* prevented LPS—induced pulmonary endothelial barrier dysfunction. 

### 2.2. Effect of TRPC6 Knockout on Altered Gene Expression in the Lungs of LPS—Treated Mice

#### 2.2.1. Differential Expression Analysis Results

To investigate the impact of *TRPC6* knockout on gene expression, we conducted an RNA—sequencing—based transcriptomics analysis in LPS— or PBS—treated *WT* and *TRPC6^-/-^* male mice. The screening criteria for identifying DEGs were *p* < 0.05 and |fold change (FC)| > 1.5, as determined by pairwise comparisons (LPS—treated *WT* mice versus PBS—treated *WT* mice or LPS—treated *TRPC6*—KO mice versus LPS—treated *WT* mice). 

**Figure 3:**
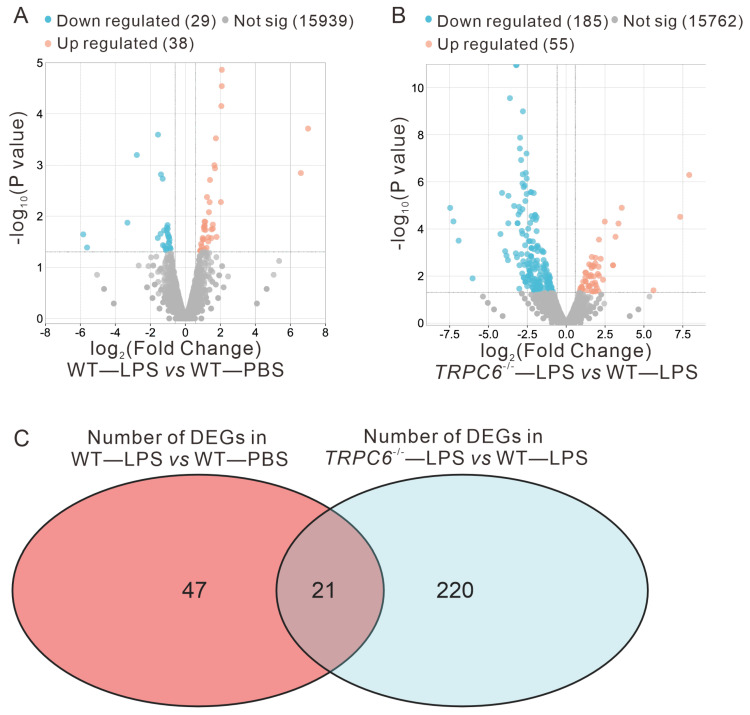
Differential analysis results. (**A**) Volcano plot illustrating the outcomes of differential expression analysis between *WT*—LPS and *WT*—PBS groups. (**B**) Volcano plot demonstrating the differential expression analysis results between *TRPC6^-/-^*—LPS and *WT*—LPS groups. (**C**) Overlapping genes identified as differentially expressed in both comparisons, i.e., *WT*—LPS vs. *WT*—PBS and *TRPC6^-/-^*—LPS vs. *WT*—LPS. Note: *p* < 0.05, |log_2_Fold Change| > 0.58.

In total, we identified 68 differentially expressed genes, with 39 upregulated and 29 downregulated genes in the *WT*—LPS vs. *WT*—PBS comparison (Figure 3A). A comparison between the *TRPC6^-/-^*—LPS and *WT*—LPS groups revealed a total of 241 differentially expressed genes, including 55 genes upregulated and 186 genes downregulated (Figure 3B). By intersecting these sets of differentially expressed genes, we found a total of 21 unique DEGs, including *APOD*, *IGHD4—1*, *EDN1*, *TIGIT*, *GBP4*, *IGTP*, *IGKV3—10*, *CD274*, *CYP26B1*, *GBP2*, *STAT1*, *GZMB*, *IRGM2*, *TGTP2*, *GBP5*, *SLAMF8*, *GBP3*, *IIGP1*, *IGKV8—27*, *GZMA*, *CCL5* (Figure 3C).

#### 2.2.2. GO and KEGG Enrichment Analysis Results

**Figure 4:**
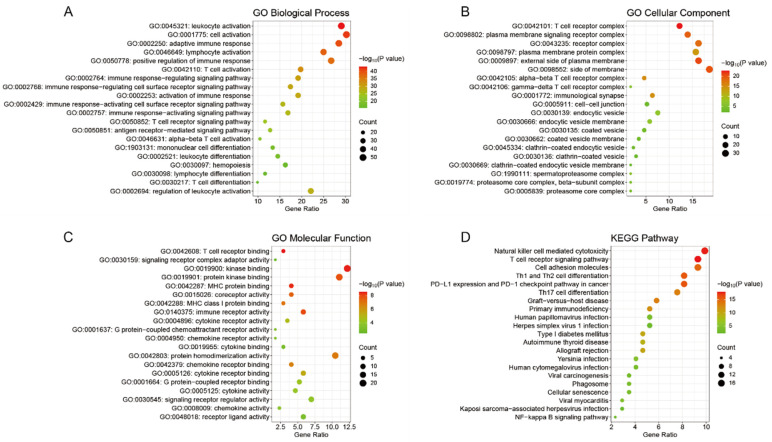
Enrichment Analysis Results. (**A**) GO Biological Processes analysis results for differentially expressed genes in *TRPC6^-/-^*—LPS vs. *WT*—LPS. (**B**) GO Cellular Components analysis results for differentially expressed genes in *TRPC6^-/-^*—LPS vs. *WT*—LPS. (**C**) GO Molecular Functions analysis results for differentially expressed genes in *TRPC6^-/-^*—LPS vs. *WT*—LPS. (**D**) KEGG Pathway enrichment analysis results for differentially expressed genes in *TRPC6^-/-^*—LPS vs. *WT*—LPS.

To investigate the key pathways underlying the observed effects associated with *TRPC6* knockout in LPS—induced ALI, we performed GO enrichment analysis and KEGG pathway enrichment analysis on the differentially expressed genes from the comparisons of *WT*—LPS vs. *WT*—PBS and *TRPC6^-/-^*—LPS vs. *WT*—LPS. The top 20 results from the Metascape platform were selected and plotted in Figure 4 and Figure S1. The GO biological processes analysis revealed that the differentially expressed genes in *WT*—LPS vs. *WT*—PBS were primarily involved in lipid polysaccharide and immune—related responses (Figure S1A). In contrast, the differentially expressed genes in *TRPC6^-/-^*—LPS vs. *WT*—LPS were mainly associated with inflammatory and immune—related processes (Figure 4A). The GO cellular components analysis revealed that the differentially expressed genes in *WT*—LPS vs. *WT*—PBS played significant roles in lysosomes, extracellular matrix, and membrane structures (Figure S1B), while the differentially expressed genes in *TRPC6^-/-^*—LPS vs. *WT*—LPS predominantly exerted their functions in lipid membrane—related regions (Figure 4B).

The GO Molecular Functions analysis indicated that the differentially expressed genes in *WT*—LPS vs. *WT*—PBS were primarily involved in molecular functions related to chemokines and cytokines (Figure S1C). In contrast, the differentially expressed genes in *TRPC6^-/-^*—LPS vs. *WT*—LPS were predominantly linked to chemokines, cytokines, and immune—related molecular functions (Figure 4C). Furthermore, KEGG pathway enrichment analysis revealed that the differentially expressed genes in *WT*—LPS vs. *WT*—PBS were mainly involved in pathways such as cytokine–cytokine receptor interaction, Toll—like receptor signaling pathway, TNF signaling pathway, and cell adhesion molecules (Figure S1D). In contrast, the differentially expressed genes in *TRPC6^-/-^*—LPS vs. *WT*—LPS were primarily associated with pathways including cell adhesion molecules, Th1 and Th2 cell differentiation, Th17 cell differentiation, and NF—kappa B signaling pathway (Figure 4D). Furthermore, lung tissues were collected from *WT*—PBS, *WT*—LPS, *TRPC6^-/-^*—LPS, and *TRPC6^-/-^*—PBS mice for subsequent analysis. 

**Figure 5:**
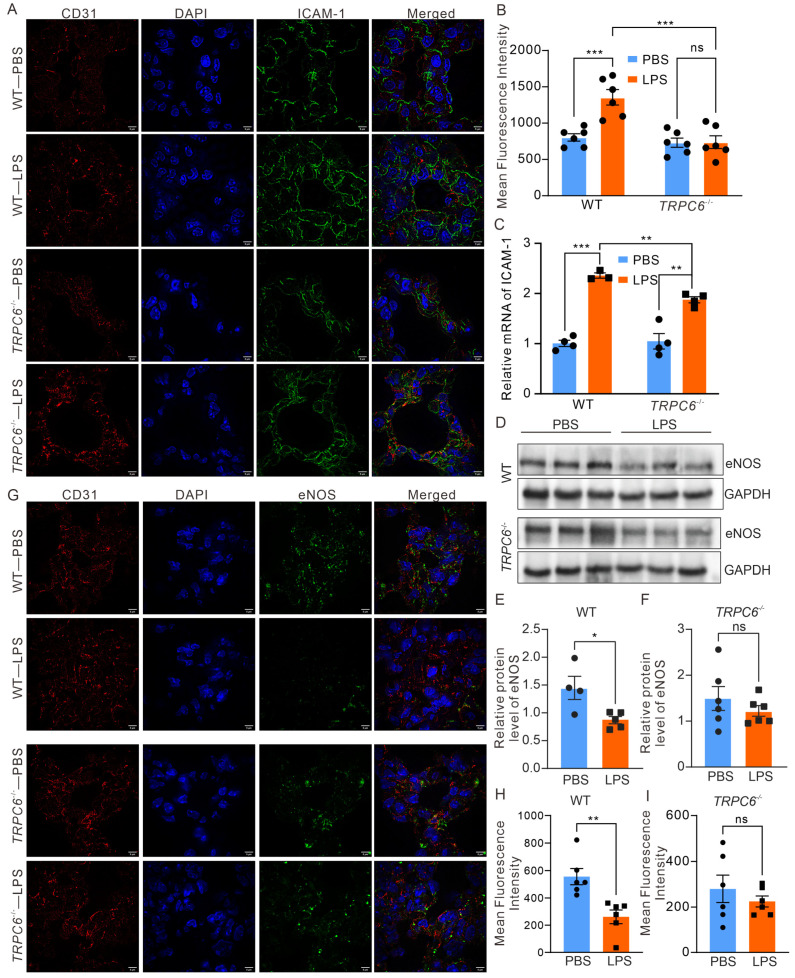
The effect of *TRPC6* deletion on eNOS expression was investigated. (**A**,**B**) Representative immunofluorescence images and the corresponding summary data of fluorescence intensity in lung tissue sections from various treatment groups. The CD31, nucleus staining, and *ICAM—1* labeling was stained by red, blue, and green fluorescence, respectively. (**C**) The mRNA of *ICAM—1* in mouse lung was analyzed. (**D**) Protein immunoblot analysis was performed to determine the expression levels of eNOS in lung tissue lysates from *WT* and *TRPC6^-/-^* mice. (**E**,**F**) Grayscale analysis was conducted to quantify eNOS protein levels. (**G**–**I**) Exemplary immunofluorescence images and the corresponding summarized data of fluorescence intensity in lung tissue sections. The red, blue, and green fluorescence are immune stained with CD31, nucleus, and eNOS, respectively. The scale bar in the fluorescence pictures represents 5 μm. Statistical significance is denoted as * *p* < 0.05; ** *p* < 0.01, and *** *p* < 0.001, ns—No significance.

The immunofluorescence staining, wherein CD31 was employed as a marker for endothelial cells in the lung tissue [15], was conducted to determine the expression levels of *ICAM—1* (Figure 5A,B). Notably, an evident enhancement in *ICAM—1* fluorescence intensity was observed in CD31—positive endothelial cells from the LPS—treated *WT* group (803.2 ± 50.45 vs. 1356 ± 106.9, *n* = 6, *** *p* < 0.001), which was mitigated in the *TRPC6^-/-^* —LPS group (738.4 ± 87.72, *n* = 6, *** *p* < 0.001). The results were further validated through qRT—PCR. A significant increase in *ICAM—1* expression was observed in the lungs of LPS—treated *WT* and *TRPC6*—deficient mice (Figure 5C). Depletion of *TRPC6* resulted in the inhibition of *ICAM—1* upregulation induced by LPS (Figure 5C).

Notably, the cell adhesion molecule pathway was commonly shared between *WT*—LPS vs. *WT*—PBS and *TRPC6^-/-^*—LPS vs. *WT*—LPS. Previous research has indicated that endothelial nitric oxide synthase (eNOS)—derived nitric oxide can downregulate the expression of endothelial surface adhesion molecules [16]. This suggests a potential link between the cell adhesion molecule signaling pathway influenced by TRPC6 and ALI, which may be closely associated with eNOS.

### 2.3. Deletion of TRPC6 Ameliorated the Downregulation of eNOS Expression in the Lungs of Mice Treated with LPS

To further confirm the association between TRPC6 and eNOS in LPS—induced ALI, we assessed the expression of eNOS in male mouse lung tissue. Our findings revealed a significant reduction in eNOS expression in the lung tissue from *WT* mice treated with LPS (Figure 5D,E, 1.45 ± 0.21 vs. 0.87 ± 0.06, *n* = 4–6, * *p* < 0.05); However, there were no significant changes in eNOS expression in *TRPC6^-/-^* mice treated with LPS (Figure 5D,F, 1.49 ± 0.26 vs. 1.22 ± 0.12, *n* = 5–6, *p* = 0.36). The results were further validated through immunofluorescence analysis, wherein CD31 was employed as a marker for blood vessels in the lung tissue (Figure 5G–I). Notably, a significant decrease in eNOS fluorescence intensity was observed in CD31—positive endothelial cells of the LPS—treated *WT* group (557.0 ± 58.71 vs. 261.7± 50.33, *n* = 6, ** *p* < 0.01), while no difference was observed between the *TRPC6^-/-^*—PBS and *TRPC6^-/-^*—PBS LPS groups (280.4 ± 60.39 vs. 225.2± 24.34, *n* = 6, *p* = 0.4156).

**Figure 6:**
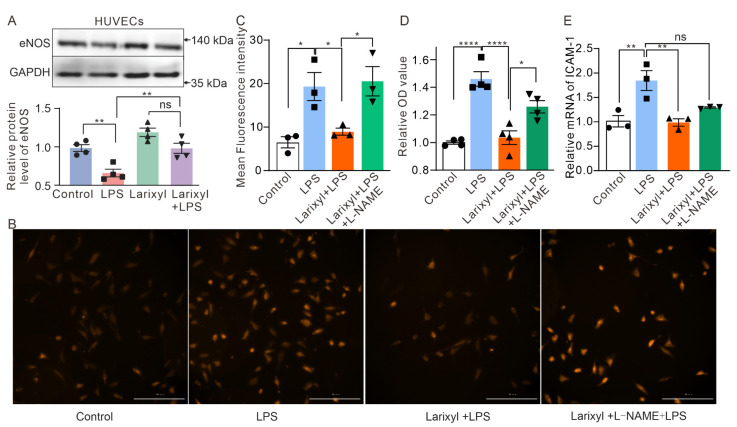
The downregulation of TRPC6 prevented LPS—induced dysfunction of HUVECs. (**A**) Protein immunoblot analysis was performed to determine the expression levels of eNOS in HUVECs treated with different drugs. (**B**,**C**) MitoSOX fluorescence probe was utilized for observing mitochondrial ROS activity in HUVECs following treatment with larixyl acetate and LPS. Scale bar, 100 μm. (**D**) Transwell was utilized for observing permeability of HUVECs following treatment with larixyl acetate and LPS. (**E**) The mRNA of *ICAM—1* in HUVECs. Statistical significance is denoted as * *p* < 0.05; ** *p* < 0.01, and **** *p* < 0.0001, ns—No significance.

Additionally, to validate the link between TRPC6 and eNOS on LPS—stimulated endothelial cells, we evaluated the expression of eNOS in HUVECs exposed to LPS with or without larixyl acetate, the TRPC6 inhibitor [17]. The results demonstrated that while there was a significant decrease in eNOS levels in the LPS group (Figure 6A, 0.98 ± 0.04 vs. 0.66 ± 0.05, *n* = 4, ** *p* < 0.01), its presence recovered when combined with larixyl acetate (Figure 6A, 0.98 ± 0.065, *n* = 4, ** *p* < 0.01). These outcomes suggest that alterations to the TRPC6 channel may impact changes to eNOS expression levels.

The dysfunction of eNOS also results in the accumulation of mitochondrial ROS (mROS) in endothelial cells [18], contributing to the disruption of endothelial barrier and lung tissue damage. Therefore, we investigated whether larixyl acetate could reduce the mROS levels in HUVECs treated with LPS using MitoSOX Red. Our results showed a significant enhancement in mROS fluorescence signals in the LPS—treated group (6.52 ± 1.27 vs. 19.34 ± 3.23, *n* = 3, ** *p* < 0.01), which remained unaltered in the presence of larixyl acetate (Figure 6B,C, 8.94 ± 0.81, *n* = 3, * *p* < 0.05). Larixyl failed to prevent the increased mROS induced by LPS in the presence of L—NAME, a specific inhibitor of eNOS (20.54 ± 3.37, *n* = 3, * *p* < 0.05). The results demonstrate that knockdown of *TRPC6* effectively suppresses LPS—induced mROS generation, which can be restored by the administration of L—NAME. The hyperpermeability of HUVECs induced by LPS was also tested by a modified Transwell assay. Consistent with the above results, inhibition of TRPC6 attenuated LPS—induced hyperpermeability, which was abrogated by the eNOS inhibitor L—NAME (Figure 6D). We also validated the effect of LPS on endothelial *ICAM—1* by RT—PCR. The treatment of LPS significantly upregulated the mRNA expression of *ICAM—1* which was effectively attenuated by larixyl (Figure 6E), consistent with findings reported by Mohammad Tauseef et al. [14]. 

## 3. Discussion

ALI is a severe pulmonary disease, and the precise mechanisms underlying this complex pathological process remain incompletely understood. Our previous study demonstrated that inhibition of TRPC6 channels significantly attenuated the dysfunction of aortic vascular endothelial cells [19]. In this study, we investigated the impact of *TRPC6* deletion on pulmonary pathological lesions induced by LPS in both male and female mice. The observed upregulation of eNOS expression in *TRPC6^-/-^* endothelial cells may contribute to the protective effect against LPS—induced lung injury, while knockdown of *TRPC6* did not affect the expression levels of TRPC1 and TRPC5.

Sex—specific differences in physiological characteristics of lung tissue play a significant role as heterogeneous factors in the development of lung diseases. Solopov et al. demonstrated that the chemically induced mouse model of lung fibrosis resulted in lower total leukocyte count and total protein content in female mouse BALF than their male counterparts [20]. In our study, female mice exhibited resistance to LPS—induced lung edema, but the neutrophil infiltration in BALF in LPS—treated mice is still significantly higher than that in sham mice. Nevertheless, *TRPC6^-/-^* mice treated with LPS did not exhibit any significant differences in neutrophil infiltration and total cell counts in the BALF when compared with *WT* female mice treated with LPS (Figure S2A–E). Both *TRPC6^-/-^* male and female mice demonstrated resistance to LPS—induced increases in total cells and neutrophils in BALF lung injury. 

When the lungs are injured, activation of ECs occurs, leading to disruption of the pulmonary microvascular barrier and altered permeability [7]. It has been reported that Ca^2+^ influx mediated by TRPC6 activates the non—muscle myosin light chain kinase (MYLK), which contributes to the hyperpermeability of lung vascular and lung inflammation [14]. The study conducted by Mohammad Tauseef et al. [14] also demonstrated the involvement of TRPC6 in ALI through the Toll—like receptor 4 (TLR4) signaling mechanism. Our study elucidated that the downregulation of TRPC6 could attenuate ALI, potentially attributed to increased expression of eNOS, and may involve the cell adhesion molecule signaling pathway mediated by molecules on cellular endothelial cells. These findings provide further insights into the role of TRPC6 in ALI disease.

It has also been reported that TRPC6 could regulate leukocyte transendothelial migration during the inflammatory response [21]. We performed transcriptome sequencing to further investigate the involvement of TRPC6 in ALI. Twenty—one common differentially expressed genes were identified in *WT*—LPS vs. *WT*—PBS and *TRPC6^-/-^*—LPS vs. *WT*—LPS. These 21 differentially expressed genes are strongly associated with acute lung injury. For example, Zhou et al. demonstrated that the regulation of the C—C motif chemokine ligand 5 (CCL5)—mediated c—Jun N—terminal kinase (JNK) and nuclear factor—kappa B (NF—κB) pathways is involved in mitigating symptoms of LPS—induced pneumonia in an in vitro model [22]. Wu et al. elucidated that modulation of signal transducer and activator of transcription 1 (STAT1) phosphorylation is associated with LPS—induced ALI, influencing vascular endothelial cell—mediated immune cell chemotaxis and adhesion [23]. Hirota et al. reported that deficiency in granzyme B (GZMB) exacerbates inflammation levels in the lungs of mice following acute lung injury [24]. Furthermore, we performed separate GO enrichment analysis and KEGG pathway enrichment analysis for the differentially expressed genes in *WT*—LPS vs. *WT*—PBS and *TRPC6^-/-^*—LPS vs. *WT*—LPS. The results of the GO enrichment analysis revealed that the differentially expressed genes in both comparisons were implicated in diverse immune—related biological processes and exhibited associated molecular functions. 

The KEGG pathway enrichment analysis revealed that both *WT*—LPS vs. *WT*—PBS and *TRPC6^-/-^*—LPS vs. *WT*—LPS were associated with multiple immune—related pathways, including cytokine–cytokine receptor interaction, Toll—like receptor signaling pathway, TNF signaling pathway, and cell adhesion molecule signaling pathway in the case of *WT*—LPS vs. *WT*—PBS. These findings suggest that the model construction of acute lung injury triggered an immune system response in the organism, which is consistent with previous literature [25]. In *TRPC6^-/-^*—LPS versus *WT*—LPS, the differentially expressed genes were primarily involved in signaling pathways such as cell adhesion molecules, Th1 and Th2 cell differentiation, Th17 cell differentiation, and NF—kappa B signaling pathway. Liu et al. reported that rosmarinic acid—4—O—β—D—glucoside can regulate inflammatory cytokine expression induced by influenza virus by downregulating Th1 cell cytokines IFN—γ and TNF—α while upregulating Th2 cell cytokines IL—4 and IL—5, thereby exerting anti—inflammatory effects in acute lung injury [26]. Additionally, several studies have suggested targeting the NF—kappa B signaling pathway as a potential treatment for LPS—induced acute lung injury [27,28,29]. These findings suggest that the modulation of the immune system by TRPC6 may also contribute to the amelioration of acute lung injury induced by LPS.

Furthermore, we observed a significant impact on the cell adhesion molecule signaling pathway in *TRPC6^-/-^*—LPS compared to *WT*—LPS. Notably, previous studies have consistently reported that TRPC6 modulates the expression of cell adhesion molecules in podocytes and other cells [30]. Therefore, the deletion of TRPC6 may also attenuate cell adhesion in the presence of LPS, thereby impeding the initiation and progression of inflammatory response in ALI. We performed RT—PCR to detect the mRNA of *ICAM—1* in both lung tissue of the ALI mouse model and LPS—treated HUVECs. The elevated *ICAM—1* in ALI mouse lung or LPS—treated HUVECs was significantly decreased by the downregulation of TRPC6 (Figure 5 and Figure 6).

The eNOS enzyme plays a crucial role in the regulation of vascular function, particularly in the modulation of vasoconstriction [31]. Numerous studies have demonstrated the significance of eNOS in the pathogenesis of ALI [5,32,33]. The expression of endothelial surface adhesion molecules is downregulated by nitric oxide derived from eNOS [16]. In our study, we observed a significant downregulation of eNOS expression in LPS—induced ALI. However, this downregulation was restored in *TRPC6^-/-^* mice (Figure 5), as well as in HUVECs (Figure 6). Furthermore, treatment with a TRPC6 antagonist significantly reduced mROS levels in LPS—treated ECs (Figure 6B,C), which was reversed by the L—NAME. These findings suggest that knockout of the TRPC6 rescues eNOS expression and exerts a protective effect on the pulmonary microvascular barrier against LPS—induced damage. However, further investigation is required to fully elucidate the TRPC6—eNOS pathways involved.

## 4. Materials and Methods

### 4.1. Materials and Reagents

LPS (lipopolysaccharides from Escherichia coli O55: B5, L118716—25 mg) was purchased from Shanghai Aladdin Biochemical Technology Co., Ltd. (Shanghai, China). PBS (G4202—500 mL) was obtained from Wuhan Servicebio Technology Co., Ltd. (Wuhan, China). 4% paraformaldehyde fixative solution (BL539A) was purchased from Beijing Labgic Technology Co., Ltd. (Beijing, China). Hematoxylin and eosin staining solution (C0133—500 mL) was obtained from Beyotime Biotech Inc. (Shanghai, China). RIPA lysis buffer (P00138) was purchased from Shanghai Beyotime Biotech Inc. (Shanghai, China). eNOS antibody (250094) was obtained from Chengdu ZEN Bioscience Co., Ltd. (Sichuan, China). GAPDH antibody (AG019—1) was purchased from Shanghai Beyotime Biotech Inc. (Shanghai, China). TRPC6 antibody (sc—515837) was acquired from Santa Cruz Biotechnology, Inc. Anti—mouse IgG, HRP (A21010) secondary antibody was purchased from Wuhan Amyjet Scientific INC. (Wuhan, China). ECL chemiluminescent enhancement reagent kit (20—500—120) was obtained from Shanghai XP Biotechnologies Co., Ltd. (Shanghai, China). MitoSOX Red and larixyl acetate were purchased from MCE (Shanghai, China).

### 4.2. Animals

Seven— to eight—week—old C57/BL6J male and female mice were purchased from Vitalriver (Beijing, China), and the TRPC6 gene knockout (*TRPC6^-/-^*) mice (C57BL/6J background) were obtained from Suzhou Saiye Biotechnology Co., Ltd. Male mice were used in this study, unless otherwise specified. The mice were housed in isolated cages under controlled environmental conditions at the Medical Research Institute of Northwestern Polytechnical University (12 h light–dark cycle, 55% ± 5% humidity, 23 °C), with free access to standard laboratory food and water. All animal studies were approved by the Medical and Experimental Animal Ethics Committee of Northwestern Polytechnical University (Approval No: 2019029) and complied with animal welfare regulations. The methods employed in this study were conducted in accordance with the approved guidelines.

### 4.3. Mouse Model of ALI

After one week of adaptive feeding, *WT* or *TRPC6^-/-^* mice were randomly divided into four groups: *WT*—PBS, *WT*—LPS, *TRPC6^-/-^*—PBS, and *TRPC6^-/-^*—LPS, with eight mice in each group. Mice were anesthetized by intraperitoneal injection of 30 mg/kg of pentobarbital sodium. The LPS group was induced for ALI by intratracheal instillation of LPS (90 μg in 30 μL PBS/mouse), while the PBS group received an equal amount of PBS via intratracheal instillation. After 24 h, the mice were euthanized, and lung tissues and bronchoalveolar lavage fluid were collected for subsequent experiments.

### 4.4. Evaluation of Lung Injury 

The total protein content, total cell counts, and classification of inflammatory cells in bronchoalveolar lavage fluid (BALF) were examined. At the end of the experiment, mice were euthanized by cervical dislocation, 500 μL PBS was instilled twice through the trachea, and BALF was collected, and this process was repeated three times. The collected BALF was used to determine the total protein content in the BALF supernatant using the BCA method, the total number of cells in the BALF was determined using cell counting plates, and finally the cells were sorted and counted using Wright–Giemsa staining solution.

Lung wet/dry weight ratio. The middle lobe of the right lung of mice was collected and rinsed with PBS. Excess surface water was removed by blotting with filter paper, and the lung tissue was then weighed using a sophisticated electronic balance to determine lung wet weight. Subsequently, the lung tissue was placed in a desiccator at a temperature of 80 °C and dried thoroughly for 72 h until a stable weight was obtained. The weight of the dried lung tissue is recorded as the lung dry weight. Finally, the lung dry/wet (W/D) weight ratio was calculated to assess the degree of pulmonary edema in mice.

Histologic examination. The collected left upper lobe was fixed overnight in 4% paraformaldehyde, dehydrated, paraffin—embedded, cut into 5 μm thick sections, and stained with hematoxylin and eosin (H&E). Images were acquired using a light microscope (Olympus) at 200× magnification. The extent of lung damage was assessed arbitrarily. The degree of lung injury was graded from 0 (normal) to 4 (severe) as follows: 0, no injury; 1, mild injury; 2, moderate injury; 3, severe injury; and 4, severe injury. Individual scores for each category and the sum of the scores of 6–8 mice in each category were calculated to determine the total lung injury score for histological assessment.

### 4.5. Cell Culture

Human umbilical vein endothelial cells (HUVECs) were purchased from Procell (Wuhan, China). After normal resuscitation, the cells were cultured in high—glucose DMEM containing 20% fetal bovine serum and 1% double antibody, placed in a constant temperature incubator containing 5% CO2 at 37 °C, and then passaged when the cells were confluent to 80%~90%, then 1/5 was added to a fresh culture flask to continue the culture, and an appropriate amount of cell suspension from the remaining cells was taken to dilute them, and then placed in a 6—well plate, and then cultured normally for 24 h. 

The cells were divided into 4 groups: control, LPS (20 ng/mL) group, LPS in the presence of larixyl acetate (10 μM) group, LPS in the presence of larixyl acetate (10 μM), and L—NAME (10 μM) group. After the cells in 6—well plates were normally cultured for 24h, they were treated with larixyl acetate or larixyl acetate + L—NAME for 1h and then with LPS for 5h.

### 4.6. Mitochondrial ROS (mROS) Measurement

MitoSOX Red mitochondrial superoxide indicator was utilized to determine mROS levels in accordance with the manufacturer’s instructions. HUVECs were processed as described in Section 4.5 and subsequently processed as follows: incubated with 5 μM MitoSOX Red reagent working solution for 10 min at 37 °C while protected from light. After washing three times with PBS, ROS fluorescence intensity was detected using a fluorescence microscope.

### 4.7. Western Blot

At the end of the experiment, 20 mg of the lower lobe of the right lung was weighed, and the total protein was extracted with RIPA lysis buffer. The protein was then quantified using the BCA method. Subsequently, 80 μg of protein was electrophoresed on an 8% denaturing polyacrylamide gel and transferred to a PVDF membrane. After blocking with a 5% BSA solution, the membrane was incubated overnight at 4 °C with anti—TRPC6 (1:800), eNOS (1:200), and GAPDH (1:1000). Then, it was incubated for another 1.5 h with horseradish peroxidase—conjugated goat anti—rabbit IgG antibody (1:10,000) and goat anti—mouse IgG antibody (1:10,000). Finally, a chemiluminescence detection reagent and imaging system were used to visualize the membranes.

### 4.8. Reverse Transcription Polymerase Chain Reaction (RT—PCR) Analysis

At the culmination of the experimental procedure, 60 mg of tissue extracted from the lower lobe of the right lung underwent quantification by weight, after which total RNA was meticulously isolated utilizing Trizol lysis buffer. Subsequently, the obtained RNA underwent reverse transcription into complementary DNA (cDNA) through the utilization of the PrimeScript™ RT Reagent Kit (Takara, RR037A, Beijing, China). Following this, polymerase chain reaction (PCR) was employed using the TB Green^®^ Premix Ex Taq™ II (Takara, RR820A, Beijing, China) for gene expression analysis.

The PCR reaction mixture, comprising 100 ng of cDNA, 12.5 μL of TB Green Premix Ex Taq II (Tli RNaseH Plus) (2×), 1.0 μL each of forward and reverse primers, and 8.5 μL of deionized water, was subjected to a thermal cycling protocol involving initial denaturation at 95 °C for 30 s, succeeded by 40 cycles of denaturation at 95 °C for 5 s, annealing at 50.5 °C for 30 s, and extension at 72 °C for 45 s. For the quantification of gene expression levels, normalization was carried out with respect to the reference gene (β—actin). The primer sequences employed for the RT—PCR analysis were as follows: *ICAM—1* (mouse): forward 5′—GTGGCGGGAAAGTTCCTG—3′, reverse 5′—CGTCTTGCAGGTCATCTTAGGAG—3′; *ICAM—1* (human): forward 5′—GGCCGGCCAGCTTATACAC—3′, reverse 5′—TAGACACTTGAGCTCGGGCA—3′; β—*actin* (mouse): forward 5′—GGCAAATTCAACGGCACA—3′, reverse 5′—GTTAGTGGGGTCTCGCTCTG—3′; *β—actin* (human): forward 5′—ATCAAGATCATTGCTCCTCCTGAG—3′, reverse 5′—CTGCTTGCTGATCCACATCTG—3′.

The determination of relative gene expression levels was accomplished employing the 2^−ΔΔCq^ method, utilizing *β—actin* mRNA as the internal control gene, facilitating a quantitative assessment of gene expression alterations.

### 4.9. Transcriptome Sequencing

Mouse lung tissue samples were obtained by dissecting the right lung, which was subsequently preserved in a liquid nitrogen tank after being washed three times with PBS. Total RNA extraction was performed using Trizol reagent (Invitrogen, Cat. No. 15596—018, Shanghai, China). Subsequently, fragmented mRNA was used as a template for reverse transcription, and cDNA fragments of approximately 370 to 420 base pairs were selected. Further, PCR amplification was conducted, and the resulting products were purified to complete the construction of the library. The constructed library was then subjected to sequencing analysis using the Illumina NovaSeq 6000 sequencing platform (Illumina, San Diego, CA, USA). The filtered reads from each sample were aligned to the reference genome (GRCm38 gene code vM25) using star v2.7.1a software, and quantification analysis was performed using featureCounts v2.0.1 software.

Differential gene expression analysis was performed using edgeR v3.28.1, with a threshold set at *p*—value < 0.05 and |fold change| > 1.5 to identify differentially expressed genes. Additionally, GO enrichment analysis, including GO biological processes, GO cellular components, and GO molecular functions, as well as KEGG pathway enrichment analysis, was conducted using the Metascape platform. These analyses allowed for the exploration and identification of enriched functional categories and pathways associated with the differentially expressed genes.

### 4.10. Evans Blue

To examine alterations in vascular permeability in response to LPS exposure, this study conducted Evans Blue staining experiments, following a methodology adapted from Smith et al. [34]. In summary, Evans Blue dye was administered via the tail vein one hour before tissue sampling. Subsequently, the lung tissue coloration was evaluated at the time of sampling to quantify the extent of vascular leakage within the lungs.

### 4.11. Modified Transwell Assay

HUVEC monolayers were cultured in Transwell inserts coated with gelatin for a period of three days. In the experimental setup, larixyl acetate or a combination of larixyl acetate and L—NAME was introduced into the upper chamber and incubated for one hour. Subsequently, LPS at a concentration of 10 μg/mL was added to activate the cells in each group. After a 5 h incubation period, horseradish peroxidase (HRP) was introduced, and the samples were incubated at 37 °C for 60 min. From the lower chamber, an equivalent volume of medium (200 μL) was extracted, and the absorbance was measured at 450 nm.

### 4.12. Immunofluorescence Assay

The immunofluorescence assay was conducted by Servicebio (Wuhan, China). The antibodies used in this experiment were CD31 (GB12063) at a dilution of 1:400, *ICAM—1* (GB11106) at a dilution of 1:400, and eNOS (GB12086) at a dilution of 1:2000. A commercial structured illumination microscope (HIS—SIM) was employed for fluorescence observation [35], and the acquired images were analyzed using ImageJ (V1.8.0.112). 

### 4.13. Statistical Analysis

Each experiment was conducted in triplicate, and the data are presented as mean ± standard deviation. Statistical comparisons between two groups were analyzed using the *t*—test, while differences among multiple groups were analyzed using one—way analysis of variance (ANOVA). The impact of LPS and *TRPC6^-/-^* on the outcome of lung injury was assessed using two—way ANOVA and the resulting parameters are presented in Supplementary Tables. GraphPad software version 9 (Prism, La Jolla, CA, USA) was utilized for statistical analysis. A *p*—value < 0.05 was considered statistically significant. 

## 5. Conclusions

In this study, we have discovered that the deletion of TRPC6 significantly mitigates LPS—induced lung edema and neutrophil infiltration in male mice. Moreover, the reduced expression of eNOS in lung tissue and endothelial cells is substantially restored upon TRPC6 knockout. Transcriptomic analysis of RNA and RT—PCR has revealed that the downregulation of cell adhesion molecules (ICAM—1) in *TRPC6^-/-^* lungs may account for their resistance to LPS—induced injury. These findings underscore the critical role played by TRPC6 in ALI, which can advance our understanding of ALI pathogenesis and offer new possibilities for its clinical treatment.

## Data Availability

The raw data supporting the conclusions of this article will be made available by the authors without undue reservation.

## Supplementary Materials

The following supporting information can be downloaded at: https://www.mdpi.com/article/10.3390/ijms242316756/s1.
