# Supplementary material for: TRPC6 Deletion Enhances eNOS Expression and Reduces LPS—Induced Acute Lung Injury
Source: Int J Mol Sci. 2023 Nov 25;24(23):16756. doi: 10.3390/ijms242316756 (PMC10706254; doi:10.3390/ijms242316756)
Supplement: Supplementary file 1 [file ijms-24-16756-s001.zip › ijms-2688601-supplementary.pdf]

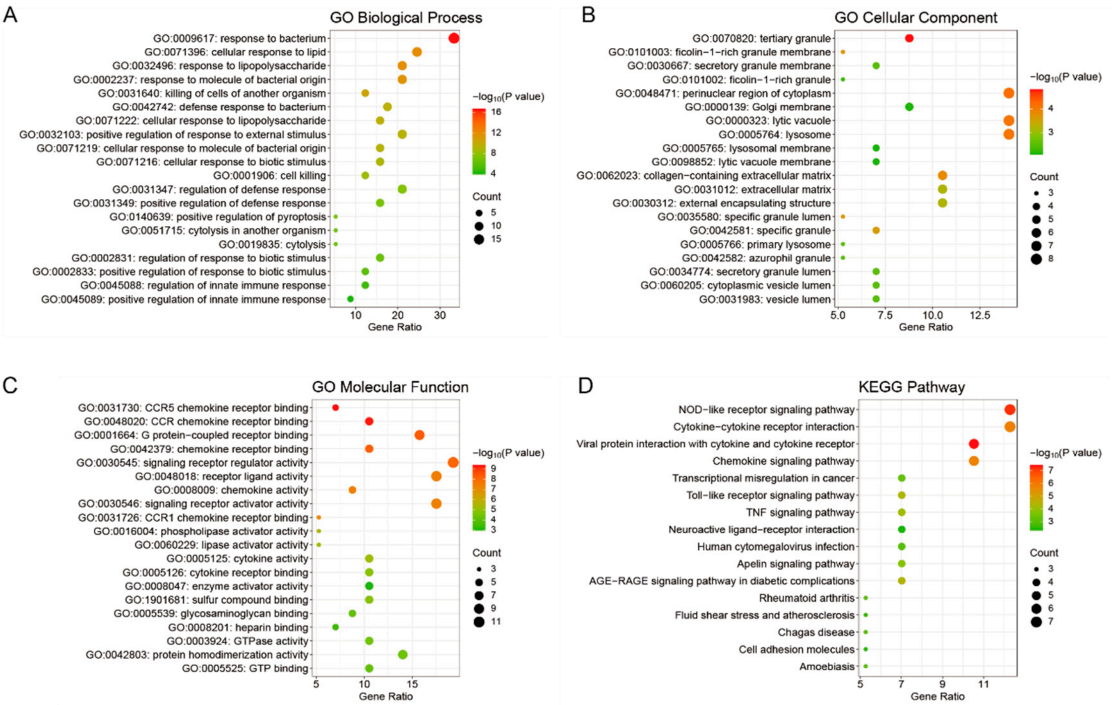

Figure S1. Enrichment Analysis Results. (A) GO Biological Processes analysis results for differentially expressed genes in WT-LPS vs WT-PBS. (B) GO Cellular Components analysis results for differentially expressed genes in WT-LPS vs WT-PBS. (C) GO Molecular Functions analysis results for differentially expressed genes in WT-LPS vs WT-PBS. (D) KEGG Pathway enrichment analysis results for differentially expressed genes in WT-LPS vs WT-PBS.

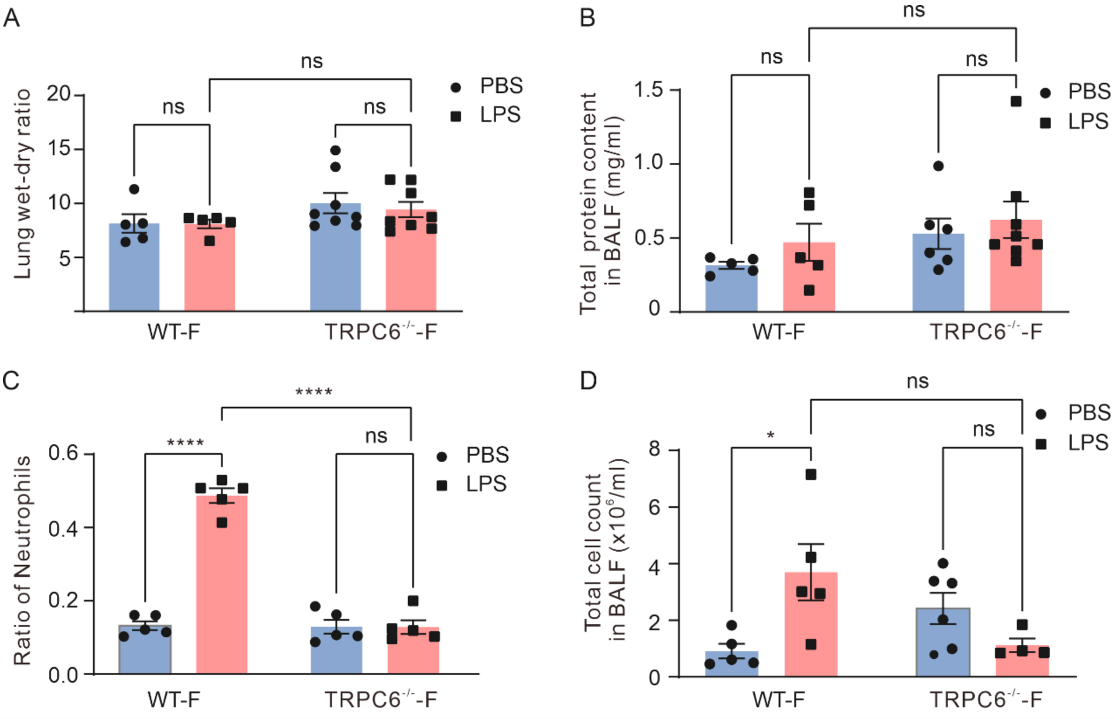

Figure S2. The impact of TRPC6 deletion on lung injury in female mice induced by ALI was assessed through measurements of lung wet-to-dry weight ratio (A), total protein content (B), neutrophil count ratio (C), and total cell count (D) in BALF. The data are presented as mean ± SEM, with statistical significance denoted as *P<0.05; **P<0.01; ***P<0.001; ****P<0.0001.
